# Consistent performance of large language models in rare disease diagnosis across ten languages and 4917 cases

**DOI:** 10.1016/j.ebiom.2025.105957

**Published:** 2025-10-14

**Authors:** Leonardo Chimirri, J. Harry Caufield, Yasemin Bridges, Nicolas Matentzoglu, Michael Gargano, Mario Cazalla, Shihan Chen, Daniel Danis, Alexander J.M. Dingemans, Klara Gehle, Petra Gehle, Adam S.L. Graefe, Weihong Gu, Markus S. Ladewig, Pablo Lapunzina, Julián Nevado, Enock Niyonkuru, Soichi Ogishima, Dominik Seelow, Jair A. Tenorio Castaño, Marek Turnovec, Bert B.A. de Vries, Kai Wang, Kyran Wissink, Zafer Yüksel, Gabriele Zucca, Melissa A. Haendel, Christopher J. Mungall, Justin Reese, Peter N. Robinson

**Affiliations:** a Berlin Institute of Health at Charité – Universitätsmedizin Berlin, Berlin, Germany; b Lawrence Berkeley National Laboratory, Berkeley, CA, USA; c William Harvey Research Institute, Barts and the London School of Medicine and Dentistry, Queen Mary University of London, London, UK; d Semanticly, Athens, Greece; e The Jackson Laboratory for Genomic Medicine, Farmington CT, USA; f INGEMM-Idipaz, Institute of Medical and Molecular Genetics, Hospital Universitario La Paz, Madrid, Spain; g Department of Pathology and Laboratory Medicine, University of Pennsylvania, Philadelphia, PA, USA; h Department of Human Genetics, Donders Institute for Brain, Cognition and Behaviour, Radboud University Medical Center, Nijmegen, the Netherlands; i Medical University of Gdansk, ul. M. Skłodowskiej-Curie 3a, 80-210, Gdańsk, Poland; j Deutsches Herzzentrum der Charité, Berlin, Germany; k Chinese HPO Consortium, Beijing, China; l Department of Ophthalmology, University Clinic Marburg - Campus Fulda, Fulda, Germany; m Trinity College, Hartford, CT, USA; n INGEM/ToMMo, Tohoku University, Miyagi, Japan; o Department of Biology and Medical Genetics, 2nd Faculty of Medicine, Charles University in Prague and Motol University Hospital, Prague, Czech Republic; p Utrecht University, Utrecht, Netherlands; q Department of Human Genetics, Bioscientia Healthcare GmbH, Ingelheim, Germany; r Institute for Maternal and Child Health - IRCCS “Burlo Garofolo” - Trieste, Trieste, 34137, Italy; s University of North Carolina at Chapel Hill, Chapel Hill, NC, USA; t CIBERER, Centro de Investigación Biomédica en Red de Enfermedades Raras, Instituto de Salud Carlos (ISCIII), Madrid, Spain

**Keywords:** Large language model, Global Alliance for Genomics and Health, Phenopacket schema, Human phenotype ontology, Genomic diagnostics, Artificial intelligence

## Abstract

**Background:**

Large language models (LLMs) are increasingly used medicine for diverse applications including differential diagnostic support. The training data used to create LLMs such as the Generative Pretrained Transformer (GPT) predominantly consist of English-language texts, but LLMs could be used across the globe to support diagnostics if language barriers could be overcome. Initial pilot studies on the utility of LLMs for differential diagnosis in languages other than English have shown promise, but a large-scale assessment on the relative performance of these models in a variety of European and non-European languages on a comprehensive corpus of challenging rare-disease cases is lacking.

**Methods:**

We created 4917 clinical vignettes using structured data captured with Human Phenotype Ontology (HPO) terms with the Global Alliance for Genomics and Health (GA4GH) Phenopacket Schema. These clinical vignettes span a total of 360 distinct genetic diseases with 2525 associated phenotypic features. We used translations of the Human Phenotype Ontology together with language-specific templates to generate prompts in English, Chinese, Czech, Dutch, French, German, Italian, Japanese, Spanish, and Turkish. We applied GPT-4o, version gpt-4o-2024-08-06, and the medically fine-tuned Meditron3-70B to the task of delivering a ranked differential diagnosis using a zero-shot prompt. An ontology-based approach with the Mondo disease ontology was used to map synonyms and to map disease subtypes to clinical diagnoses in order to automate evaluation of LLM responses.

**Findings:**

For English, GPT-4o placed the correct diagnosis at the first rank 19.9% and within the top-3 ranks 27.0% of the time. In comparison, for the nine non-English languages tested here the correct diagnosis was placed at rank 1 between 16.9% and 20.6%, within top-3 between 25.4% and 28.6% of cases. The Meditron3 model placed the correct diagnosis within the first 3 ranks for 20.9% of cases in English and between 19.9% and 24.0% for the other nine languages.

**Interpretation:**

The differential diagnostic performance of LLMs across a comprehensive corpus of rare-disease cases was largely consistent across the ten languages tested. This suggests that the utility of LLMs in clinical settings may extend to non-English clinical settings.

**Funding:**

10.13039/100000051NHGRI 5U24HG011449, 5RM1HG010860, R01HD103805 and R24OD011883. P.N.R. was supported by a Professorship of the Alexander von Humboldt Foundation; P.L. was supported by a National Grant (PMP21/00063 ONTOPREC-ISCIII, Fondos FEDER). C.M., J.R. and J.H.C. were supported in part by the Director, 10.13039/100006132Office of Science, 10.13039/100006151Office of Basic Energy Sciences, of the US 10.13039/100000015Department of Energy (Contract No. DE-AC0205CH11231).

**Research in context**

**Evidence before this study**

Large language models (LLMs) have been shown to be effective at providing differential diagnostic support by returning ranked lists of candidates when prompted with a summary of the case. Most published analyses of the performance of LLMs in this task have used English-language prompts. Most medical texts available for LLM training are in English. An important question is whether LLMs are able to respond accurately to differential diagnostic queries in other languages. Several studies have compared performance between English and other languages such as Japanese, Chinese, German, and Arabic, but at the time of this writing, no published study was available that compared the performance of an LLM on thousands of rare-disease cases in multiple languages.

**Added value of this study**

We present a large-scale study on 4917 GA4GH Phenopackets with 360 rare genetic diseases. We used translations of Human Phenotype Ontology terms and a templated approach to translate other clinical data available in the Phenopackets to create equivalent prompts in Chinese, Czech, Dutch, French, German, Italian, Japanese, Spanish, and Turkish. We showed that there was only a small difference between the performance of English and each of the other languages.

**Implications of all the available evidence**

Available evidence suggests that LLMs are able to respond to differential diagnostic prompts with similar accuracy in English and nine other languages. This result has important implications for deploying LLM-based differential diagnostic solutions around the globe.

## Introduction

Large language models (LLM) are being carefully investigated in the medical domain owing to their linguistic and problem solving capabilities. These artificial intelligence (AI) models are pre-trained by ingesting large amounts of unlabelled data in order to learn to generate coherent text, thereby opening up an array of possibilities in disparate aspects of clinical practice. Moreover, LLMs have been shown to encode latent medical knowledge and to be able to carry out deductive reasoning, which would make them candidate assistance tools in hard-to-diagnose, complex clinical cases.^1^

The majority of medical literature and therefore the LLMs’ relevant training data is in English. According to CommonCrawl,^2^ an open repository of web crawl data that can be used to estimate the distribution of internet data used by LLMs for training, 43% of available web pages are in English (version CC-MAIN-2024-51). The percentages for the other nine languages in our study range from 1.0% (Czech) to 5.4% (German). While the precise training data for most LLMs are not publicly disclosed, estimates suggest a high proportion of English language content.^3^ Thus, more material is available for training in English than in any other language, which may imply an expectation of LLMs to achieve higher performance in English, as suggested from previous studies.^4^, ^5^, ^6^ This clearly would have implications for their integration in clinical practice, as well as on the topic of AI fairness in the health domain. Few studies about multilinguality of generalist LLMs in biomedicine have been carried out, most of which tested the performance of a LLM on medical licencing exams or standardised medical questions in one non-English language or one language as compared to English (e.g., Japanese,^7^ in-context enhanced Chinese,^8^ German,^9^ and Arabic^5^).

Roughly 25% of patients with rare diseases go 5–30 years without a diagnosis, and 40% of initial diagnoses are wrong.^10^ There are at least 10,000 RDs,^11^ and the diagnostic yield of genomic sequencing is still low (25–50%).^12^ Therefore, the differential diagnosis of RDs represents a challenging task with which to evaluate the capabilities of LLMs.

LLMs have shown promise in supporting the differential diagnosis of RDs in English,^13^ yet most of the earth's population does not use English as their first language and clinical practice is carried out in diverse languages across the world. In this paper, we assess the relative performance of LLMs in RD differential diagnostics by creating a large multilingual set of published patient descriptions. Thereby we address limitations of previous studies that used simulated/synthetic patients or small cohorts and provide a comprehensive analysis of the relative performance of a leading general purpose LLM and a recent medically fine-tuned model in ten different languages.

## Methods

### Overview of study

We conducted a comparison of the ability of LLMs to support genetic differential diagnostics across several languages. We analysed 4917 case reports from the literature and generated LLM prompts in ten languages. We then directed two LLMs, openAI's GPT-4o and the medically fine-tuned Meditron3-70B,^14^ based on Meta AI's open source Llama-3.1-70B-Instruct, to return a ranked list of possible diagnoses for each case. The Mondo disease ontology,^15^ which contains numerous synonyms for each disease, was used to map the diagnoses returned by the LLM to a unique standardised medical vocabulary, to which we applied our automatic ontology-aware scoring.^16^ The rank of the correct diagnosis in the output was compared across languages in order to assess to what degree the LLM's performance is prompt-language dependent. This study is reported according to the TRIPOD-LLM reporting guideline.^17^

### Human Phenotype Ontology internationalisation

The Human Phenotype Ontology (HPO) provides a standardised vocabulary of 19,034 terms that describe the phenotypic abnormalities of human disease. Version 2024-12-12 was used for this study. Additionally, the HPO provides a comprehensive corpus of phenotype annotations (HPOA) that form computational models of 8333 rare diseases. HPO applications include genomic interpretation for diagnostics, gene-disease discovery, machine learning (ML) and electronic health record (EHR) cohort analytics.^18^ The HPO Internationalisation Effort comprises language-specific working groups that have translated HPO term labels and in some cases synonyms and definitions from English into other languages.^19^ For the current project, we used ten languages that have extensive coverage of HPO-term translations, namely Chinese, Czech, Dutch, French, German, Italian, Japanese, Spanish, and Turkish translations. All translations are freely available (see data availability section). Information about the number of available translated terms present in HPO is found in Supplemental Table S1. Translations are created by or confirmed by human experts before inclusion in an HPO release.

### Structured data from case reports: phenopackets

The Global Alliance for Genomics and Health (GA4GH) Phenopacket Schema is a standard for sharing phenotypic, genetic and clinical information.^20^ The Phenopacket Schema obtained International Standard Organisation approval as ISO 4454:2022. Each Phenopacket is a clinical vignette about one individual with representations of phenotypic abnormalities using HPO terms, as well as a specification of the disease diagnosis and other information.

The Phenopackets used in this project were selected from the Phenopacket Store version 0.1.19, an openly available collection of Phenopackets manually curated from published case reports.^21^ It contains a total of 6668 Phenopackets representing 475 diseases and 423 disease genes. We restricted our analysis to the subset of Phenopackets in the Phenopacket Store for which translations of all associated HPO terms exist in the nine languages mentioned above. This yielded a dataset of 4917 Phenopackets (1590 females, 1826 males and 1500 unspecified) from 706 PubMed IDs, comprising 326 causative disease genes, 360 diseases, 2899 alleles, 2525 unique HPO terms and an average of 14 HPO terms per patient.

### Prompt generation

**Fig. 1:**
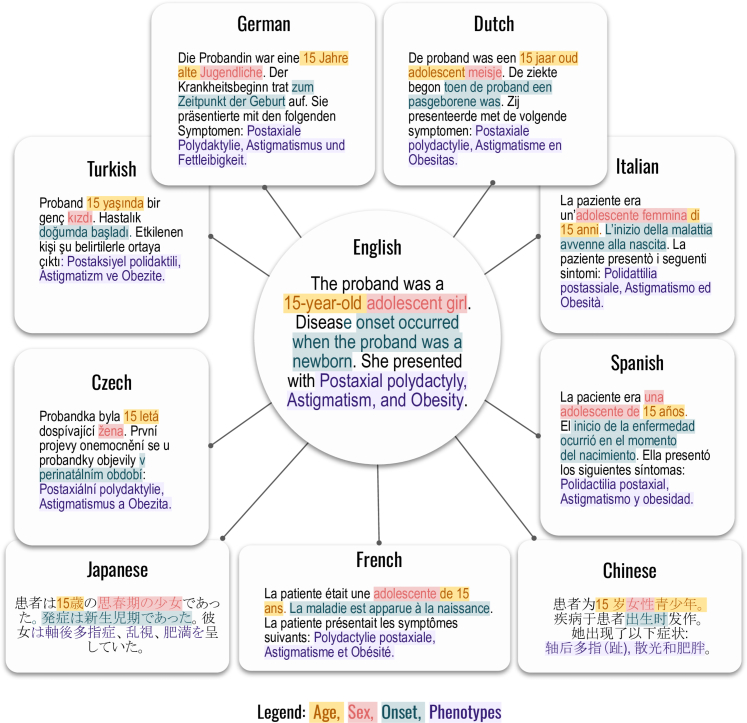
**Templated system for generating prompts using translation of the HPO into 9 languages.** An excerpt of one prompt is shown. Words representing age, sex, onset, and phenotypes are colour-coded as indicated.

Phenopackets comprise a hierarchical structure that is typically stored as a JSON file. We developed a strategy to create narrative prompts from each Phenopacket by a templating system implemented in a Java application called phenopacket2prompt.^22^ Each template consists of constant texts (such as the header that instructs the models to return a differential diagnosis), and a series of templates to represent the age and sex of the individual represented by the Phenopacket as well as phenotypic abnormalities that were observed or excluded. If available, the age of onset of the disease or specific manifestations is recorded. The templating system involves vocabulary for describing the individual in each of the languages. HPO terms in each of the languages are substituted into the corresponding templates. The translators of each of the nine languages are physicians or medical researchers, and an example of the part of a prompt describing a patient is shown in Fig. 1. The correctness of the translation templates was confirmed by review of 54 simulated cases that were output in each language using permutations of ages, sexes, as well as observed and excluded HPO terms. The diagnosis and the genetic information were not included in the prompts. At the end of the constant section, we state that the case is a genetic disease and we request the LLMs to return an ordered list of candidate diagnoses, giving an example output. The example output is always given in English and in non-English languages we explicitly instruct the LLM to return the differential diagnosis in English. To evaluate the effect of this choice, we prompted GPT-4o with 100 randomly chosen cases in Italian, German and Spanish, but giving the example output of the prompt in the respective language and dropping the request for an English reply. We then manually evaluated and scored GPT-4o's output differential diagnoses in 100 cases in these three languages (see Supplementary Figure S1). An example Phenopacket with associated full prompt in English and its translation into the nine languages is available in Supplementary Tables S3 and S4.

### Grounding and scoring

The queries to GPT-4o were carried out through its API between November 22nd, 2024 and May 20th, 2025. The knowledge cutoff claimed by openAI is October 2023 for all GPT-4o models, including gpt-4o-2024-08-06, the version we used. We used the default parameters (temperature of 1, with no token length limitation) which roughly amounted to 22.5 million input tokens (corresponding to a cost of about US $112) and 2.1 million output tokens (about US $42). Meditron3's base model is Llama-3.1-70B-Instruct, which has a knowledge cutoff of December 2023 and officially supports English, German, French, Italian, Portuguese, Hindi, Spanish and Thai, though its training data contains a broader collection of languages.^23^ We downloaded and ran the model on a local HPC cluster with 2 NVIDIA A100 80 GB SXM4 GPUs. The run took approximately 18 h, with the maximum output token length of 2048 (and otherwise the model's standard parameters: temperature of 0, seed of 1234, and the Meditron-specific parameter of “task_type” set to mcq).We instructed the LLM to reply in the form of free text (e.g., Marfan syndrome), rather than a corresponding ontology term identifier (e.g., MONDO:0007947), because the task of returning identifiers may be prone to so-called “hallucinations”, where plausible-sounding yet wrong answers are given.^24^^,^^25^ We leveraged our pheval.llm pipeline to parse the LLM response for free-text candidate diagnoses, to identify the corresponding ontology identifier, and to rank genetic subforms of a clinical disease as equivalent (e.g., Loeys-Dietz syndrome and any of its six genetic subforms were regarded as equivalent for the purposes of assessing correctness of the differential diagnosis). PhEval.llm is a freely available plugin for the PhEval framework and leverages Monarch Initiative LLM tooling.^16^^,^^26^^,^^27^

With this, we could computationally score the responses of both GPT and Meditron for all Phenopackets in all ten languages, corresponding to 98,340 differential diagnoses with more than 544 thousand candidate diseases (of which 8273 unique guesses). Finally, we computed the number of correct diagnoses found at the top of the differential diagnosis (“Top-1”), within the first three candidate diseases (“Top-3”), and likewise for “Top-10”. This procedure was carried out for all ten languages.

### Role of the funding source

The funding sources had no role in study design, data collection, data analysis, data interpretation, writing of the report, and decision to submit.

### Ethics

Ethical approval was not required.

## Results

In this work we leveraged translations of the Human Phenotype Ontology into Chinese, Czech, Dutch, French, German, Italian, Japanese, Spanish, and Turkish to test the relative performance of GPT-4o and Meditron3-70B in differential diagnostic support. To do so, we leveraged GA4GH Phenopackets, representing the clinical data (phenotypic abnormalities and diagnosis) for 4917 patients with 360 diseases drawn from 706 publications. We generated a programmatic templating system that generated a narrative text using templates to represent the age, sex, and age of onset of disease together with observed and excluded phenotypic features (Fig. 1).

Results are counts of correct diagnoses at a given rank in the differential diagnosis, shown in Table 1 for GPT-4o and in Table 2 for Meditron3 aggregated at “Top-1”, “Top-3”, and “Top-10”. The “Not ranked” column shows the number of cases in which the LLM reply did not contain the correct diagnosis. Additionally, the “No Diagnosis” column indicates the number of cases in which the returned text could not be parsed as a differential (for example, “I'm sorry but based on the information provided, I cannot return a confident diagnosis”). In no case did we observe a correct result beyond rank 10, so that the sum of the last three columns in Tables 1 and 2 is always 4917, the total number of cases. Depending on the language and model, between 1.6% and 16.0% items in the differential could not be successfully grounded (i.e., the Mondo term corresponding to the diagnosis could not be identified; see Supplemental Table S2). Excluding Japanese and Chinese (the two languages not based on Latin scripts) for Meditron3, the grounding failures for both models lie in the range of 1.6%–6.2%.

**Table 1:** Results of differential diagnosis by GPT-4o by language.

Language	Top-1	Top-3	Top-10	Not ranked	No diagnosis
English	978	1328	1532	3384	1
Spanish	941	1355	1560	3357	0
Czech	880	1240	1396	3495	26
Turkish	978	1373	1544	3265	108
German	938	1290	1439	3377	101
Italian	851	1314	1498	3416	3
Chinese	909	1338	1401	3510	6
Dutch	1006	1357	1503	3391	23
Japanese	790	1245	1352	3318	247
French	918	1338	1524	3388	5

Numbers indicate counts, not ranked includes grounding failures but not instances of refusal by GPT to deliver a diagnosis, which is counted separately in the rightmost column “No Diagnosis”.

**Table 2:** Results of differential diagnosis by Meditron3-70B, see caption of Table 1 (however, we did not observe any refusal to reply and therefore drop the “No Diagnosis” column).

Language	Top-1	Top-3	Top-10	Not ranked
French	751	1094	1229	3688
Spanish	755	1117	1358	3559
Japanese	833	1181	1293	3623
German	752	1088	1309	3608
Dutch	782	1118	1379	3538
Chinese	810	1105	1176	3741
Turkish	670	978	1178	3735
Czech	659	1006	1180	3735
English	756	1028	1141	3776
Italian	767	1089	1310	3607

**Fig. 2:**
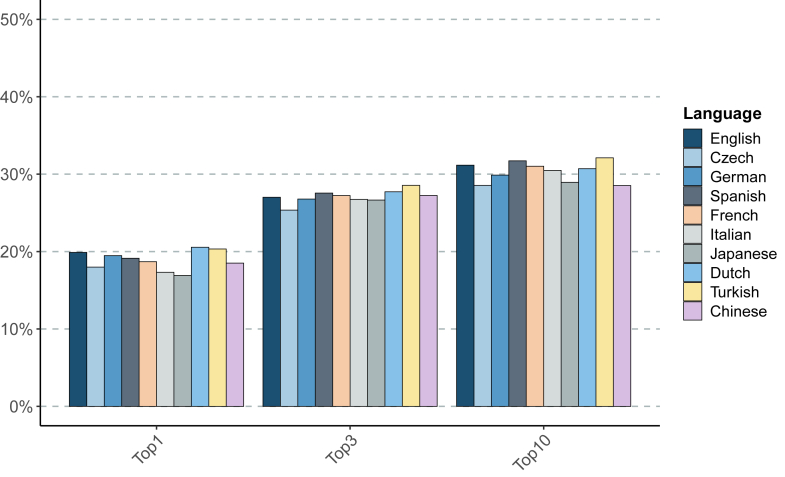
**Differential diagnostic performance of GPT-4o in English, Chinese, Czech, Dutch, French, German, Italian, Japanese, Spanish, and Turkish.** The percentage of cases in which GPT-4o place the correct diagnosis in rank 1 (Top-1), within the top three ranks (Top-3) or within the first ten ranks (Top-10) is shown.

**Fig. 3:**
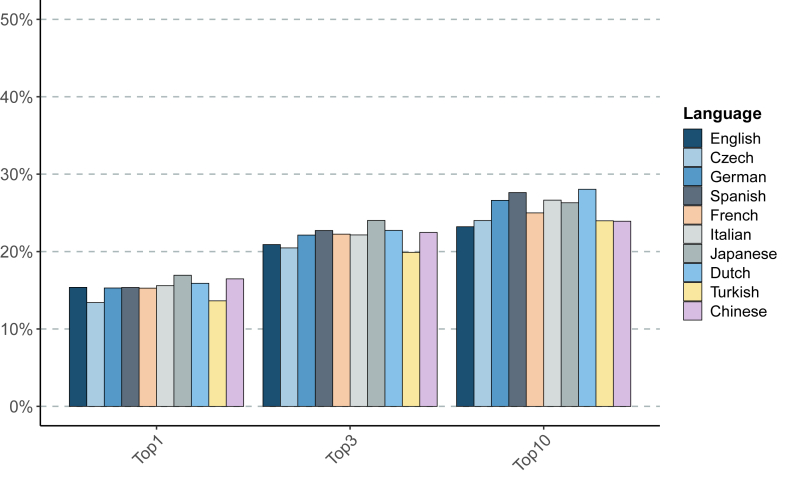
**Differential diagnostic performance of Meditron3-70B in English, Chinese, Czech, Dutch, French, German, Italian, Japanese, Spanish, and Turkish.** The percentage of cases in which Meditron3-70B place the correct diagnosis in rank 1 (Top-1), within the top three ranks (Top-3) or within the first ten ranks (Top-10) is shown.

In Figs. 2 and 3 we show frequencies obtained as Top-N divided by the total number of cases excluding those in “No Diagnosis”. To test for statistical differences between ranks in different languages we used SciPy's ‘stats’ package to perform a Kruskal-Wallis^28^ H-test. We obtain as a statistic H = 30.8 and p-value = 0.0003, for GPT-4o and H = 55.5 and p-value of 10^−8^ for Meditron3, indeed indicating statistically significant differences between the languages.

In openAI's GPT-4o English was the third best performer at Top-1 and Top-10. At Top-1, there were 19.9% of correct cases in English while other languages ranged from 16.9% to 20.6%. At Top-3, the relative spread decreases, with the correct diagnosis among the Top-3 candidates in the differential diagnosis 27.0% of cases for English, compared to the range 25.4%–28.6% for the other languages. This comprises a *relative* difference of *at most* 6% for Top-3 with respect to English, and at most 9% for Top-10, where English scored 31.2% while other languages lie in the range 28.5%–32.1%.

The medically fine-tuned Meditron3-70B shows a worse overall performance (though this may be largely attributable to the likely much smaller size of the model) with quite some variability at Top-10. English scored 15.4% at Top-1 while other languages ranged between 13.4 and 16.9%, and the highest scorer at Top-10 was Dutch with 28.0%, with English lagging behind at 23.2%, the lowest of all. The largest *relative* difference with English at Top-3 consists of 13% and is with Japanese.

## Discussion

We prompted the GPT-4o and Meditron3 language models with 4917 RD cases in ten different languages. All languages in this study constitute at least ∼1% of the CommonCrawl, which is a proxy for the amount of relative internet data available in a given language, a reflection of the language-specific data available for training. For these ten languages we have shown that GPT-4o and Meditron3 are able to perform differential diagnostics for RD with similar performance. This would be surprising if LLMs only used language-specific models to answer queries because most of their training data and of relevant medical literature is in English. Although the performances do vary, our result suggests that this model can generalise medically relevant knowledge derived mainly from English language texts in order to answer queries posed in (at least) the nine non-English languages tested.

The creation of a large set of realistic vignette-like prompts originating from real cases and translated in multiple languages overcomes limitations of previous diagnostics studies with LLMs, such as the utilisation of extensive and unrealistically long case reports,^29^ the usage of simulated/synthetic patients,^30^^,^^31^ the usage of comparably small cohorts,^29^, ^30^, ^31^ and the difference in style and length of real clinical notes coming from different countries.^31^

Our study has several limitations. We used the same zero-shot prompting strategy for each language and did not attempt to improve performance using more sophisticated strategies such as chain of thought or retrieval augmented generation approaches.^32^ Our evaluation made use of lists of phenotype terms, rather than narrative clinical notes, and thus may not reflect challenges or nuances relating to individual languages. Additionally, we only tested two models, and were only able to test a selection of relatively widely used European and Asian languages native to roughly 2.3 billion people. Therefore, further research will be needed to determine the potential utility of LLMs in clinical settings where other languages are spoken. We chose the ten languages for this study because the translations had been previously created by groups collaboration with the HPO project. We invite colleagues from other countries to contact us in order to create HPO resources in additional languages. We are not able to precisely assess to which extent failures in mapping free text to Mondo identifiers were responsible for the slightly worse performance of some languages in our testing. We noticed Meditron3 had a significantly higher grounding failure rate for non-Latin scripts, since sometimes the model would ignore our request for an English reply. Our results with GPT-4o and Meditron3 suggest that in principle it is possible to train an LLM to leverage medical knowledge that is predominantly recorded in English to support differential diagnostics in other languages.

Although it is difficult to measure the extent of data contamination bias in LLM, data contamination is known to affect the results of benchmark evaluations of LLM on a variety of tasks.^33^ It is likely that GPT and Meditron3 had access to some of the published clinical data used to perform the evaluation, and thus the evaluation results may not reflect what to expect on new data. However, we know of no other dataset with several thousand cases available in ten languages and that is also unpublished (and thus not subject to data contamination bias).

The ability of LLMs to carry out any diagnostics of complex cases in different languages is notable considering they are general purpose language models predominantly trained with English data. Both OpenAI and Meta AI claim to have significantly increased the multilingual capabilities with respect to their previous models.^23^^,^^34^ The vast amount of data used by LLMs for training can lead to data contamination that may overestimate the performance to be expected for new data.^35^ Therefore, the performance measured in our study may not generalise. Future research will be required to understand if generalisation performance is dependent on prompt language. Finally, we instructed the LLMs to return the diagnoses in English, which was necessary to be able to perform grounding for our computational analysis. Our comparison of the results in Italian, German, and Spanish for 100 cases with diagnoses returned in either English or the other language did not show a consistent difference between the approaches (Supplemental Figure S1).

Despite the interest in using LLMs to support clinical care, LLMs are not currently ready for autonomous decision making.^36^ Before widespread application of LLMs in English or other languages in clinical care, it will be necessary to develop strict guidelines about accurate and ethical use of LLMs.

Consistent performance across languages has implications for the implementation of these models in clinical practice across the globe. Many people in low- and middle-income countries (LMICs) have limited access to healthcare services.^37^ As LLMs become increasingly proficient in supporting differential diagnosis and related domains such as bedside consultation question answering and addressing questions from the general public,^38^ there is a great potential to improve care for people in LMICs by supplementing existing systems with LLM-driven services. It would be desirable to offer such services in local languages, especially for consumer-facing applications. Future work will be required to assess performance of LLMs in LMICs (all languages assessed in our study are from high-income countries).

## Contributors

L.C., J.R., and M.G. created the pheval.llm python code with input from J.H.C., E.N. and Y.B. L.C. wrote the first draft of the paper in close collaboration with P.N.R. The phenopacket2prompt Java application was written by P.N.R. and L.C. who also wrote the Italian prompts and coordinated the language specific developers of the prompts. M.H. conceptualised the ontology translational strategy and Phenopackets. L.C. and M.G. conducted the experiments on the HPC cluster. N.M. developed much of the technical infrastructure around translation management and is the main liaison for the HPO translation community. P.N.R. and J.R. led the overall study. C.M. devised the Mondo evaluation approach and advised on the use of LLMs.

S.C. coded the Chinese prompts; G.W. is part of the development of Chinese HPO; K. Wang confirmed the Chinese translations; D.D. coded the Czech prompts; M.T. provided Czech translations and confirmations; A.S.L.G. coded the Turkish prompts and helped with Turkish translations; Z.Y. confirmed and provided Turkish translations; D.S. suggested and confirmed German translations; P.G., M.S.L. and G.J.P. confirmed the German translations; K.G. confirmed the French translations; G.Z. provided the Italian translations; J.N., J.A.T.C, M.C., and P.L. provided the Spanish translations; K. Wissink coded the Dutch prompts; A.J.M.D. and B.B.A.d.V. provided the Dutch translations; S.O. provided the Japanese translations. L.C., J.R., and P.N.R. had full access to and verified the underlying data. All authors read and approved the final version of the manuscript and take responsibility for the decision to submit the publication. All of the data used in this study is publicly available.

## Data sharing statement

HPO translations: https://obophenotype.github.io/hpo-translations/ 4917 phenopackets and corresponding prompts in English, Chinese, Czech, Dutch, French, German, Italian, Japanese, Spanish, and Turkish, together with all our results at DOI: https://zenodo.org/records/14907503.

All code is publicly available at: pheval.llm: https://github.com/monarch-initiative/pheval.llm, phenopacket2prompt: https://github.com/P2GX/phenopacket2prompt, ontology access kit: https://github.com/INCATools/ontology-access-kit.

## Declaration of interests

MH is a co-founder of Alamya Health. D.S. received a payment by Sanofi for a presentation in a continuing medical education course about AI and rare diseases.
